# Experiences with the Recent Epidemic of Rabies in Buffalo, N. Y.1Read before the Pathological Society of Rochester, January 17, 1901.

**Published:** 1901-05

**Authors:** Ernest Wende

**Affiliations:** Health Commissioner, Buffalo, N. Y.


### BUFFALO MEDICAL JOURNAL.

VOL. XL.—LVI. MAY, 1901. NO. 10.

### Original Communications.

### EXPERIENCES WITH THE RECENT EPIDEMIC OF RABIES IN BUFFALO, N. Y.[1]

BY ERNEST WENDE, M. D.,
Health Commissioner, Buffalo, N. Y.

AN important fact to be gathered from history, is the liability of human beings to be attacked by diseases of a contagious nature, transmitted through the agency of inferior animals. These diseases often present such unusual features of interest, and severity as to engage the serious attention of the local health authorities, and alarm the public generally. And none more so than rabies, on account of its cruel character, distressing anxiety, mortality, its ever present source—the dog, and the unfortunate dispute as to its actual existence. It is a much easier and more popular task to dilate concerning the habits and good traits of this inseparable companion, than to describe the peculiar morbid manifestations which can not be considered apart from or independent of the other mammalia that have been infected through his bite. It must be viewed in conjunction with man, if we would hope to form a just, enlarged and correct idea of its true significance and pathological affinities. It is one associated with misconception and ignorance, which entails a large amount of needless and woeful suffering, and even death.

It is not my intention to make a thorough analysis of this interesting subject, nor to discuss its antiquity, extent or origin, but merely to record a few facts and experiences that are especially connected with the epidemic of rabies, circumscribed within certain bounds in and about the City of Buffalo, N. Y., and now practically under control.

---

1. Read before the Pathological Society of Rochester, January 17, 1901.

I wish to accentuate that the malady was first observed in November, 1898, in a pug-dog, owned by Edward Sherk, residing in the Kenmore section of the city, followed by a cow being attacked, the property of Charles Hand, and, later, by a horse of White's livery stable, on Jersey Street, the latter dying and the former being killed.

These were followed by a number of isolated cases, scattered throughout the city, at varying intervals, until June, 1899, when a stray dog acting strangely, appeared in Alden, N. Y., and bit a dog belonging to Ira Mann, which subsequently went mad, ran amuck biting several dogs, and among them, it was surmised, the large Newfoundland owned by George Cook. He was of a playful and tractable disposition. He had never strayed from his master's threshold. He was everybody's crony, the pet of the neighborhood, the willing comrade and frolicsome playmate of the village school children. It would almost seem that he was too gracious an animal, in the process of time, after catching the infection to have become frenzied and furious, and to have wandered miles from home, snapping at everything alike, whether animate or inanimate, and biting three men, two horses, four cows, and God knows how many dogs.

Of the men, J. Getz was attacked and severely bitten in the arm, near Wende Station, a distance of four miles from the home of this once affectionate animal. Otto Seihl was bitten in the hand, at Looneyville, a mile or more further on, and S. J. Walter, likewise, was bitten in the hand in the Township of Lancaster, at a remoteness of at least ten miles.

The Department of Health to arrest this terrible infection of these men intrusted to its care did not hesitate to advise them to take immediate advantage of the treatment to be obtained at the Pasteur Institute, New York, for, with human life at stake, it saddened the heart to think that chance should rule, where law ought to reign. These, then, were our first cases to leave Buffalo for the special treatment of this unique and mischief-making complaint.

Of the horses bitten, one died; of the cows, three; while the dogs were mostly killed, leaving but a few to be securely housed for demonstration and observation of facts; however, only to see them grow uneasy, develop the disease and die.

Somewhat later, at Derby, N.Y., a singular tramp dog also turned up, behaving in an uncustomary manner, and, before being shot, bit seventeen dogs and two cats, all of which were promptly destroyed. These two cases mentioned must suffice to illustrate the deadly work that can be perpetrated by a single mad dog, for similar instances have been so frequent with us that it would be tiresome to try to enumerate them all.

Verification of the true nature of the malady was made in numerous cases by Drs. Bissell and Carpenter, bacteriologists of the Department of Health of Buffalo, with the result of uniformly finding the diagnosis to be correct. Further verification was had in portions of the brain of dogs sent to Professor Moore of Cornell University and the Bender Laboratory at Albany, notably in one case—the Roquet case—where the dog presented but vague evidence of the malady. Direct inoculations upon fox terrier dogs were made jointly by Dr. John Wende, veterinarian, and Dr. Carpenter, of the Health Department, from rabid dog brains, with the result of developing the malady on an average of the twenty-third day.

In a communication, to me, from Deputy Commissioner W. C. Patrick, of the State Department of Agriculture, I am able to give the following illustrative cases of the epidemic in the rural districts. One vagrant dog bit one calf, one cow and possibly others. Calf and cow both died with pronounced symptoms of rabies. A second vagrant dog bit one calf and three cows, of which the calf and two of the cows died—undoubted signs of rabies. A third vagrant dog bit one horse and one cow, both died, typical rabies. A fourth vagrant dog bit one horse, two hogs and one cow; all died with rabies.

These animals belonged respectively to LaSalle Tabor, Frederick Boldt, Marilla; James Vine, Alden; W. J. Romeing, Abel Seeley, Christina Frazer, B. J. Stockwell, Lockport.

These dogs were all acting strangely when observed, were aggressive, and bit without provocation, and attempted, though failed, to bite many other animals. The animals bitten, and which died, were particularly examined and studied, and their malady diagnosticated as rabies, by Dr. Kelley, State Veterinarian, Albany, N.Y., and Dr. Anderson Crowforth, Lockport, N. Y.

The question of municipal control for the restriction of the spread of rabies is most important, presenting many peculiar aspects. The experience of the Department of Health in securing legislation in the interest of public health shows that such procedures can not be obtained without strong opposition from certain classes, some, undoubtedly, well-meaning but not well-informed, others well-conned but not well-disposed.

Ordinances relating to sanitation, contagious disease, and the regulation of the practice of medicine, have all been born with opposition that has ultimately fallen into merited oblivion, but in none has there been such unreasonable, violent antagonism as towards measures directed to restrain the possibility of injury from dogs. It would appear by those referred to that the possible discomfort of dogs was of more moment than the protection of human beings. Muzzles were objected to as cruel; leashing and chaining as injurious to their health, while evasion of the dog ordinance, regulating the action of animals most likely to be infected, is practised and justified from a sense of false sentiment or morbid heroism. It was obvious that no laws, however good, could be substituted for good citizenship, and it was evident that the public is much to blame for this apathy, carelessness, opposition and cussedness.

The presence of a multitudinous dog population in a community serves no good purpose; their diminution and restriction is indicated and should be accomplished. They should be permitted only under restraint of safeguards. It should be the policy of the authorities of all large cities to have regulations of such character as would cause not only a decided decrease in the number of worthless curs usually prevalent, but would limit their character and ownership to those most desirable, thus benefiting the community and the dogs themselves, which, in relation to rabies, would mean less material, less menace, and greater facility of control. Unless every care is taken, each individual case is liable to start an epidemic. Only those who make a special study of this question can be fully impressed with its importance. The extreme ignorance of the public generally, in this matter, is a fact admitted by all capable of judging on the subject, and is, indeed, manifested by evidence of the most abundant and notorious kind. The most false and most absurd notions are entertained respecting the whole subject and respecting the means capable of removing it. The prevalence of rabies in large municipalities is a grave danger, not only to the well-being of the community itself, but to all the surrounding territory, and equally pernicious, if not more so, are the so-called anti-rabic wiseacres who trade upon opposition, which, unfortunately, obtains to such an extent in these days, and is, in my opinion, unmitigated insincerity of the most detestable description. Here, as elsewhere, it is apparent that their mind can scarcely ever escape from the conventional thraldom in which it has been nursed. They naturally oppose and restrain the unselfish efforts of the sanitarian in his attempts to blot out sickness, sorrow and suffering, by dodging the truth and working upon the ignorant to deceive the public, and to increase their own consequence. Like the witches and impostors that have always held competition with physicians, caring nothing for the safety and health of the public, but continually striving to induce it to swallow falsities for facts I have often wondered if it ever occurred to them that such misery and death might be entirely prevented.

At any rate, and in all events, it is the duty of an honest mind, on attaining conviction of an error, to abandon and to explain it, even although the truth that is sure to succeed it, may, as yet, be seen only darkly, or be entirely hidden.

Therefore, every city, in dealing with dogs, should be provided with efficient rules, regulations, and ordinances, and demand their rigid enforcement, the features of which should embody: (1) Police supervision, registration, tagging, a large license fee and penalties; (2) capture and destruction of all dogs at large, not so registered and tagged; (3) muzzling and leashing all dogs when at large; (4) distribution, by police, to registered dog owners, of cards, educational in character,giving information concerning care of dogs, rabies, ordinances, penalties; (5) periodical police house to house census to determine that such regulations are in force; (6) stringent quarantine against all localities known to contain this disease; and additionally during periods of epidemics; (7) a large force of extra dog-catchers to raid the streets, catching, for destruction, all unmuzzled, unleashed and unlicensed dogs; (8) police instruction to destroy such animals, whenever found practicable, by shooting; (9) destruction of all dogs, cats or other animals bitten under even suspicious circumstances; (10) Immediate cleansing and disinfection, under supervision, of stables and premises where animals have been harbored suffering from rabies; (11) the destruction, by fire or through cleansing with hot soap suds and disinfection, of kennels, bedding and feeding utensils; (12) the skinning of carcasses of animals dying from rabies or suspected to have died from rabies should be strictly forbidden; (13) all carcasses of animals that have suffered from rabies, real or suspected, should be rendered harmless by cremation; (14) prohibition to cure any animal suffering from rabies, or being suspected of suffering from rabies, without official permission; (15) the transportation, if ever necessary, of animals having or suspected of having rabies, should only be performed in a closed vehicle or receptacle.

By this combined system of raiding and police action, rapid extermination is effected. Both methods are considered actually necessary on account of the dog-catching system being so possible of evasion—owing to the fact that the whereabouts of these officials spread like wildfire, dogs are secreted, and efficiency is thereby crippled. Therefore, in order to gain speedy protection, to succeed in suppressing an epidemic of this nature, the community at large must graciously express a willingness to accept the indicated procedures.

Briefly, the prominent clinical characteristics of the Buffalo epidemics were: (1) History of altered behavior of the animals, they showing irritability, restlessness, changed disposition; (2) desire to swallow foreign bodies, wood, stone, glass, dirt, grass, etc.; (3) a tendency to stray; in the majority of instances, the dogs left their homes, wandering away for one or more days, an act unknown in their previous lives, and returning in a bad condition—sick.

Of all the symptoms, in the furious stage, none were so constant, characteristic and diagnostic as the alteration in voice; almost every dog manifested this evidence of paralysis of the vocal chords in the hoarse howling bark, changing into a high pitched falsetto-like note.

All were characterised by biting and snapping at imaginary objects to furious aggression towards anything before them—the floor, bucket of water, sticks, any article, in fact, within their reach. Many would hold on so tenaciously to objects held before them, as to be able to be lifted by their hold, and moved from one place to another. A marked symptom observed in cases of the furious form, and one to which attention has apparently not been called or observed, and which may become a strong differential diagnostic point, was the absence of any inclination, on the part of dogs, to shrink or retire from impending blows or attacks.

Ordinarily, a dog, approached with a club in the attitude of a coming blow, recedes, dodges, and evades it. In dogs, however, with this malady, blows could be aimed directly towards their eyes, face or head, without their giving any evidence or appreciation of the impending danger to them of possible injury or pain. This was so noticeable as to be commented upon by even non-professional persons, and was demonstrated over and again. Of course, when objects were held before them, or were coming towards them, they would frequently attack them viciously, blindly, but, between the paroxysms of fury, when quiet, dogs approached with a club aggressively as stated would pay no attention, the act being unappreciated. This is not the case with all well dogs who, under such circumstances, depending upon their dispositions, appreciate the movement and either endeavor to evade or retire from it or attack it. This feature, noticed in rabies, I believe to be characteristic and, if experience in this epidemic is a criterion, it will be found in the future when looked for.

Another symptom noticed and, heretofore undescribed, when spasms of muscles of deglutition were violent, was a pouching out of the throat, visible externally. When present in this degree, these spasms were followed by relaxation of the sphincters and involuntary defecation and urination.

At the dog pound from the inception of the epidemic up to December, 1900, there were destroyed nearly 6,000 dogs, among which were 95 furious and 38 dumb cases. From April 1, 1899, to December 31, 1900, the Department of Health investigated 315 cases of persons bitten by dogs. Of the 41 persons bitten by rabid dogs in the city, four died in the agonies of hydrophobia. A synopsis of their cases seems pertinent on account of their rarity.

William Barry, living in the Township of Cheektowaga, at the city boundary, had a mixed breed collie watch dog. The child of one of his near neighbors, Tresselt, eight years of age, in passing the gate on his way home from school, was, without provocation, bitten by the dog which, at the time, was loose in the yard. On Barry's return to his home, and the fact of the biting being reported to him, he searched for the dog; found him secreted under the barn, and in securing him was bitten slightly on the hand himself. The dog was killed and taken to the bone-yard, when, on opening his stomach, it was found to contain sticks, stone, grass and other foreign bodies. No further consideration was, apparently, given to the matter of the biting beyond ordinary medical attendance at the time.

Eighty-one days later, however, the Tresselt boy was taken sick and died on the fourth day in great suffering, his illness presenting a picture of rabies in the human being. The symptoms were general malaise followed by rapidly developing irritability of the nervous system; mental depression, wakefulness, apprehension, strained or anxious expression of the face, hoarse voice. By the second day these features were intensified and mild convulsions occurred, particularly of the throat. Fluids, water, and the like, on being attempted to swallow precipitated these spasms. During this period there was a slight temperature which gradually increased to 102°.

From the unusual nervous irritability, expression, spasms, intensified in throat and excited by attempts to drink, the family physician, Dr. Danser, without any knowledge of a dog having bitten the child, made the diagnosis of hydrophobia, when inquiry revealed the fact that eighty-one days previously he had been bitten by Barry's dog. The boy died on the fourth day in a state of coma which had been preceded by intensification of all the symptoms, particularly those relating to deglutition, nervous irritability and mental apprehension. So intense was his nervous condition that the boy had to be held on his mother's lap constantly.

Peculiar features of the case, as observed by Dr. Heath and also myself, were the spasms of the throat, the mental condition of apprehension and expression of face. This was peculiar, it being one of intense anxiety and fear on a pallid countenance with blue lips and lividity over the molar bones which, in contrast with the sunken, staring, glassy eye, presented a picture of singular aspect.

The death of the boy brought a realisation of the possibilities of the case to Barry, who was despatched, without delay, to the Pasteur Institute in New York, for treatment, where he died under similar circumstances, the disease developing in him just two days later than in the boy.

Autopsy of the Tresselt boy, which was performed by Dr. W. H. Heath, of the Health Department, showed boy well developed; some emaciation; rigor mortis slight; skin cyanosed, particularly in dependent part of the lips; mucous membrane of the mouth and throat more especially swollen, dark colored and congested; larynx and large bronchi reddened, swollen, an excess of tenacious mucus present; lungs moderately congested; heart in diastole contained death clot, and large vessels filled with dark, heavy blood; stomach normal; liver very full of blood; kidneys normal; bladder contained small quantity of urine, no albumin; brain—intense congestion over the cortex, most marked posteriorly and over medulla extending down and fading away over six or seven inches of upper cord; on section, red punctated points of engorged capillaries present; membranes edematous and not adherent; autopsy showed no pathognomonic lesions; death due to exhaustion and heart failure.

Case III.—A. F., a strong vigorous, exemplary young man, while attending a dance, a stray fox terrier dog wandered into the dance hall acting peculiarly. Fisher attempted and succeeded in throwing the dog out, but was bitten in the hand in so doing, as was also a second young man who had attempted it just previously. Neither of them paid any attention to the incident, further than to have their bites cauterised and properly dressed at the Fitch Hospital. On or about the thirty-six day he was taken ill with symptoms of an indefinite character, but which soon developed into a typical case of hydrophobia, when he was removed to the Buffalo General Hospital, dying there in the greatest agony after an illness of but four days.

The facts of his illness were that he was taken sick with gradual increasing irritability of the nervous system, headache, general depression—physical and mental—and tremor, to such an extent that he was scarcely able to hold the tools of his trade at which he was endeavoring to work in spite of his condition.

Dr. Edward Meyer was called and sent his assistant who reported he did not know what ailed the man, but that he was peculiarly sick. Dr. Meyer, who then saw him, reports that he found him suffering from intense headache, a peculiar nervousness, "that the bed he was in looked as if fourteen different kids had played in it," anxious expression, some spasms or twitchings, and difficulty with the throat. On being given fluid to swallow, a teaspoonful of milk produced spasms of muscles of deglutition. Upon this suspicious circumstance, the doctor learned that some time previously he had been bitten by a dog under suspicious circumstances and the diagnosis of hydrophobia made and the man removed to the Buffalo General Hospital.

I saw this case at the Hospital where he presented a fearful picture. Convulsions were present in such a degree that the subcutaneus tissues were ruptured, permitting a general cellular emphysema around the neck and thorax, giving the man a most hideous appearance. It was necessary to have him tied down to the bed and otherwise restrained. During the interval between some of the spasms he asked to be loosened which was done, when it required the combined strength of several men to restrain him. While being carried from one room to another, to insure quiet, he was seized with one in the hall in which he died. During this time and in the intervals of spasms, he was conscious, rational, spoke. He expectorated profusely. His inability to swallow was absolute and his temperature elevated.

During the time he was in the hospital, his case was observed by a number of prominent physicians, all of whom diagnosticated the case as typical, also considered it one of the most cruel deathbed scenes in their experience. The other man who was bitten disappeared, but was finally found with some difficulty and sent to the Pasteur Institute, at New York, for treatment, and it is gratifying to note that he escaped the fate of his companion.

The fourth case of death from rabies in human being was in the person of Mariana Dolitski, of 375 Peckham Street. She was under the care of Dr. Emil Lustig, one of our prominent East Side physicians, and died, after a three days' illness, from typical hydrophobia. The death certificate of this case, as in the others, are on file in the office of the Registrar of vital statistics.

The procedure recommended by the Department of Health and followed during this epidemic was, locally, the application of a ligature around the parts where feasible, and thorough cauterisation with nitrate of silver—carbolic acid, antiseptic dressing, free drainage; bleeding should be encouraged. It is not believed that any local application would positively prevent the development of rabies in a person bitten by a rabid dog, but is urged as prevention of local septic condition and as contributing to allay mental apprehension, nervousness.

There is no general treatment, save that of prophylaxis by what is known as the Pasteur treatment, with which you are all familiar. Its success has justified its use in every instance where indicated. In the presence of the malady itself in man, the indications are to prevent irritation, prolong life, allay spasm. How futile the endeavor to fulfill these indications we all know. Cocaine to the fauces, and chloroform for spasm. Morphia and curare have been recommended but, in reality, exert no influence over the course of the disease.

Those who dispute the existence of this disease and its fatal consequences, likewise assail the honesty of Pasteur's great work, the usefulness of the Pasteur Institutes and the integrity and even commercial honesty of those who operate them.

Our experience and the deduction best drawn from it is briefly as follows: There were sent to New York, for this treatment, a total of thirty-seven persons. These individuals were all bitten by dogs pronounced rabid after careful examination by Dr. W. H. Heath of the Department of Health, a graduate of the University of Pennsylvania, and lately of the Marine Hospital Service. The doctor brought to this work the proper training, education and reputation, and further, a strong personal interest, all of which is referred to as evidence of the correctness of the deductions and weight of opinion. During this epidemic, many prominent physicians were induced to make personal examination for themselves of the nature and character of the malady, and the various phases of it present in different cases. Of these, many were known to be dog owners, skeptical, or non-believers in hydrophobia, and it is gratifying to note, that, with that honesty characteristic of the true medical man, there was no difficulty nor hesitation in recognising the malady, nor in acknowledging their previous erroneous views.

One of these gentlemen, Professor Charles Cary of the University of Buffalo, in undergoing this change of opinion, considered it his duty to exhibit a case of the affection to the medical class of the University and give a clinical lecture thereon.

The subjoined list contains the names, addresses and dates of admission of patients sent from Erie County to the Pasteur Institute for treatment in 1899:

J. Getz, Wende Station, July 20.

O. Seihl, Looneyville, July 22.

S. J. Walter, Lancaster, July 22.

T. B. Barry, Sloan, August 8.

J. Barrett, August 13; C. M. Dake, August 14; H. Roquett, August 25; G. Gerome, ———; R. Fairchild, September 7; Dr. F. A. Crandall, September 5; W. C. Hebard, September 8; E. H. Hadley, October 8; Miss D. Page, October 8; R. Page, October 8; E. Brotherton, October 8; B. Archangelo, October 8; W. Reese, October 17; Miss L. Montgomery, October 17; H. Williams, October 20; Mrs. B. Hines, October 28; W. H. Burke, November 3; E. O'Leary, November 6; J. Herb, November 24; Miss L. Sorgel, December 13, all of Buffalo.

M. Schwartz; Hamburg.

In 190c were the following: W. C. Wickham, January 9; J. Klahn, January 18; P. W. Miller, January 23; C. J. Wade, January 28; F. Lanck, January 28; W. Wiener, February 17; C. Zimmerman, March 1; G. Wende, April 27; O. Mertz, April 27; Freddie Stewart, August 31; John P. Hantges, September 10; B. S. Erskine, November 8, of Buffalo.

This epidemic demonstrated the fact that, at least, in this section, popular misconception concerning certain features of hydrophobia continue to exist to a large extent and had to be contended with, viz: that after dog bites, in event of the dog becoming rabid, the person bitten would become likewise affected; that dogs should be killed at once to prevent themselves from becoming liable to the malady, and singularly, no reference was made, at any time, in press or letters to the press, to the superstition and curative properties of the mad-stone.

To the writer, it would appear superfluous to seriously present facts substantiating the existence of the malady in dogs and its communication to man, were it not that a number of well-meaning, no doubt, but misguided persons exist who take the opposite view to the detriment and injury of their fellow-beings.

When consideration is given to the fact that governments abroad recognise the malady and make provision for it, that it has been the subject of investigation and demonstrated to be a malady of the most horrible possibilities, and by men of the highest ability and undoubted integrity, that it is considered in all classifications of disease, in all courses of medical education, in all text-books, it seems incredible that there can be found any to dispute the question. As the anti-vaccinationists seek, for other reasons than the true one, to explain the subjugation of smallpox, so do these persons seek, by the most untenable way in the light of facts to explain this malady. They can not or will not see the simple fact that dogs get a fatal malady, inflict fatal wounds upon dogs and man with fatal results, and that it is possible to practically and successfully avert these consequences in man through a treatment which is based upon the correctness of views held by medicine concerning it, they are so strenuous to deny in this connection.

471 DELAWARE AVENUE.

